# The Genetic and Epigenetic Mechanisms Involved in Irreversible Pulp Neural Inflammation

**DOI:** 10.1155/2021/8831948

**Published:** 2021-03-08

**Authors:** Xiaoxi Xi, Yihong Ma, Yuzhen Xu, Anthony Chukwunonso Ogbuehi, Xiangqiong Liu, Yupei Deng, Junming Xi, Haitong Pan, Qian Lin, Bo Li, Wanchen Ning, Xiao Jiang, Hanluo Li, Simin Li, Xianda Hu

**Affiliations:** ^1^Department of Stomatology, Northeast Petroleum University Affiliated Hospital, Fazhan Road, High Tech District, 163000 Daqing City, Heilongjiang Province, China; ^2^Department of Neurology, Graduate School of Medical Sciences, Kumamoto University, Kumamoto 860-0811, Japan; ^3^Department of Neurology, Shanghai Tenth People's Hospital, Tongji University School of Medicine, No. 301 Middle Yanchang Road, Shanghai, China; ^4^Department of Physics, University of Münster, Wilhelm-Klemm-Str. 9, 48149 Münster, Germany; ^5^Laboratory of Molecular Cell Biology, Beijing Tibetan Hospital, China Tibetology Research Center, 218 Anwaixiaoguanbeili Street, Chaoyang, Beijing 100029, China; ^6^Department of Stomatology, Daqing Oilfield General Hospital, Zhongkang Street No. 9, Saertu District, 163000 Daqing City, Heilongjiang Province, China; ^7^Department of Prosthetics, School of Stomatology, Second Affiliated Dental Hospital of Jiamusi University, Hongqi Street No. 522, Jiamusi City, Heilongjiang Province, China; ^8^Department of Stomatology, South District Hospital, Daqing Oilfield General Hospital Group, Tuqiang Fourth Street No. 14, Hong Gang District, Daqing City, Heilongjiang Province, China; ^9^Department of Conservative Dentistry and Periodontology, Ludwig-Maximilians-University of Munich, Goethestrasse 70, 80336 Munich, Germany; ^10^Stomatological Hospital, Southern Medical University, 510280 Guangzhou, China; ^11^Department of Cranio Maxillofacial Surgery, University Clinic Leipzig, Liebigstr. 12, 04103 Leipzig, Germany

## Abstract

**Aim:**

To identify the critical genetic and epigenetic biomarkers by constructing the long noncoding RNA- (lncRNA-) related competing endogenous RNA (ceRNA) network involved in irreversible pulp neural inflammation (pulpitis).

**Materials and Methods:**

The public datasets regarding irreversible pulpitis were downloaded from the gene expression omnibus (GEO) database. The differential expression analysis was performed to identify the differentially expressed genes (DEGs) and DElncRNAs. Functional enrichment analysis was performed to explore the biological processes and signaling pathways enriched by DEGs. By performing a weighted gene coexpression network analysis (WGCNA), the significant gene modules in each dataset were identified. Most importantly, DElncRNA-DEmRNA regulatory network and DElncRNA-associated ceRNA network were constructed. A transcription factor- (TF-) DEmRNA network was built to identify the critical TFs involved in pulpitis.

**Result:**

Two datasets (GSE92681 and GSE77459) were selected for analysis. DEGs involved in pulpitis were significantly enriched in seven signaling pathways (i.e., NOD-like receptor (NLR), Toll-like receptor (TLR), NF-kappa B, tumor necrosis factor (TNF), cell adhesion molecules (CAMs), chemokine, and cytokine-cytokine receptor interaction pathways). The ceRNA regulatory relationships were established consisting of three genes (i.e., LCP1, EZH2, and NR4A1), five miRNAs (i.e., miR-340-5p, miR-4731-5p, miR-27a-3p, miR-34a-5p, and miR-766-5p), and three lncRNAs (i.e., XIST, MIR155HG, and LINC00630). Six transcription factors (i.e., GATA2, ETS1, FOXP3, STAT1, FOS, and JUN) were identified to play pivotal roles in pulpitis.

**Conclusion:**

This paper demonstrates the genetic and epigenetic mechanisms of irreversible pulpitis by revealing the ceRNA network. The biomarkers identified could provide research direction for the application of genetically modified stem cells in endodontic regeneration.

## 1. Introduction

Pulpitis, as the neuroinflammation of the sensory trigeminal afferent axons in the dental pulp tissue, is accompanied by the pain induced by the stimulation of the pulp nerve fibers [1]. As a dynamic immune-inflammatory disease, the balance between the inflammatory and regenerative responses in the diseased pulp determines the clinical outcome, for example, from healthy pulp to reversible pulpitis, to irreversible pulpitis, and until pulp necrosis and pulp death [2]. Irreversible pulpitis is of high therapeutical relevance, as it is characterized by lingering pain that is featured by thermal stimuli, spontaneous pain, and pain at night [3]. Root canal therapy (RCT) based on pulpotomy remains the only choice for irreversible pulpitis; however, RCT can cause the teeth to be more brittle and thus more easily fractured [4]. Given this complication of RCT, researchers are attempting to use a combination of mesenchymal stem cells, biomaterial scaffolds, and growth factors to preserve dental pulp and achieve the neurovascularization of pulp tissue based on methods of modern tissue engineering. Nevertheless, pulp regeneration approaches face many challenges such as lifespan and diffusion of growth factor, as well as degradation of biomaterial. To overcome all of these challenges, genetically modified stem cells have been increasingly investigated and have also been shown to achieve better efficacy compared to using stem cells alone [5]. Since genetically modified stem cell transplantation could be promising in endodontic regeneration, it is therefore essential to have a deep understanding of the genetic and epigenetic mechanisms involved in the pathology of pulpitis.

With the advent of gene detection techniques, the genetic and epigenetic mechanisms have been shown by microarray and sequencing datasets [6, 7] to play a critical role in the immune-inflammatory response and repair response of pulpitis. As is well known, a messenger RNA (mRNA) as a protein-coding RNA can be targeted by multiple noncoding RNAs such as microRNAs (miRNAs) and long noncoding RNAs (lncRNAs) [8, 9]. Based on the competing endogenous RNA (ceRNA) hypothesis proposed by Salmena et al., lncRNAs harboring miRNA response elements (MREs) and mRNAs can compete with each other by binding to a shared miRNA, thereby acting as molecular “sponges” and inducing translational repression at the posttranscriptional level [10]. Since the ceRNA network has been demonstrated to be involved in many inflammatory conditions and cancers [11], this network is possibly also involved in pulpal inflammation and should therefore be investigated. The bioinformatic techniques integrate all of the expression profiling datasets available to the public and allow the identification of critical biomarkers involved in the ceRNA network to be possible. Up until now, there is only one study available that examined this issue; however, there are differences in terms of study designs and processes, thereby obtaining quite distinct results [12].

Therefore, the current study is aimed at identifying many genetic and epigenetic biomarkers, including significantly enriched pathways of differentially expressed genes, critical genes involved in the protein-protein interaction network and the ceRNA network, critical miRNAs and lncRNAs involved in the ceRNA network, and transcription factors involved in the TF-mRNA network. The identification of these biomarkers will be helpful for the genetic modification of stem cells and will benefit pulpal regeneration and the shift from irreversible pulpitis to reversible pulpitis.

## 2. Methods

### 2.1. Procurement of Datasets

The microarray datasets regarding irreversible pulpitis were searched from the GEO of the NCBI database [13]. The genetic datasets could be investigating mRNA expression profile or noncoding RNA expression profile. The inclusion criteria of datasets were established as follows. (1) The study design of the included datasets should be established as two groups, including normal pulp tissue as the control group and inflamed pulp tissue as the experimental group. (2) The samples were taken from the adults (18 years or older) presenting for endodontic treatment with no evidence of periapical pathoses (i.e., radiolucency, swelling, and pressure sensitivity) and no previous pulp therapy (i.e., pulp capping). (3) Normal pulp tissues in the control group of the included datasets were collected from healthy third molars or teeth extracted for orthodontic purpose. Inflamed pulp tissues in the experimental group of the included datasets were extracted from teeth diagnosed with irreversible pulpitis in accordance with the endodontics diagnoses system from the American Association of Endodontists. (4) The sample size for each group in the included datasets should be more than three. The exclusion criteria of datasets were established as follows: (1) the datasets which had the different study design; (2) the datasets which took the pulp samples from the teeth with periodontitis/incompletely developed roots; (3) the datasets which took the pulp samples from the patients who had a compromised immune system or those who were taking medications known to influence the immune response; (4) the sample size for each group in the included datasets was less than three. According to the inclusion and exclusion criteria mentioned above, two datasets (GSE92681 and GSE77459) were therefore obtained.

### 2.2. Procurement of miRNA-mRNA and miRNA-lncRNA Interaction Data

The human's experimentally validated miRNA-target interaction pairs' data that have been validated by experiments were downloaded from three databases: TarBase (version 6.0) [14], miRTarBase (version 4.5) [15], and miRecords (version 4) [16]. The human's experimentally validated miRNA-lncRNA interaction pairs' data were downloaded from the starBase (version 2.0) database [17].

### 2.3. Differential Expression Analysis

Regarding the dataset GSE92681, the probe sequences were reannotated because the corresponding gene symbols of probes cannot be obtained. The lncRNA and mRNA data were obtained from the platform of the GSE92681 dataset after annotation. The differential expression analysis was performed by using the Linear Models for Microarray data (limma) R/Bioconductor package [18] to identify the differentially expressed genes (DEGs), and differentially expressed lncRNAs (DElncRNAs) were identified between irreversible pulpitis samples and control healthy pulpal samples. The gene and lncRNAs that had the required cutoff criteria (*p* value < 0.05 and a ∣log_2_ fold change (FC) | >1) were considered as DEGs and DElncRNAs. The Venn diagram was used to visualize the overlapped and union DEGs identified by two datasets.

### 2.4. Functional Enrichment Analysis of DEGs

The DEGs overlapped by two datasets (GSE77459 and GSE92681) were used for the functional enrichment analysis in order to explore the regulated biological processes and signaling pathways that are involved by these DEGs. The functional enrichment analysis was performed by using clusterProfiler package in the Bioconductor package [19]. The functional terms with *p* value < 0.05 were regarded to be significant.

### 2.5. Construction of Protein-Protein Interaction (PPI) Network

To comprehensively analyze the functions of DEGs involved in the entire biological network of pulpitis, the union DEGs identified from two datasets were used for the PPI network analysis instead of only taking the overlapped intersection DEGs. The interacting genes of these DEGs were downloaded from HPRD [20] and the BioGRID database [21]. The visualization of a PPI network was performed by using Cytoscape software [22]. Several topological features (i.e., degree, average shortest path length, betweenness centrality, closeness centrality, clustering coefficient, and topological coefficient) of the nodes (protein) in this PPI network were calculated by using CytoNCA plugin in Cytoscape software to screen hub genes. The top 20 genes were selected from this network, and their topological features were listed.

### 2.6. Weighted Gene Coexpression Network Analysis

To further analyze the functions of interacting genes in the pathogenesis of pulpitis, the weighted gene coexpression network was constructed by using weighted gene coexpression network analysis (WGCNA). The genes with *p* value < 0.05 were selected, and the expression profile data of these genes were obtained. The significant gene modules were selected, and genetic interactions within each module were investigated. Based on the HPRD and BioGRID database, the PPI network of the selected significant gene modules was constructed, respectively. The top 25 gene nodes in these PPI networks were calculated and listed.

### 2.7. Functional Enrichment Analysis of DElncRNAs

Based on the GSE92681 dataset, the coexpression status of DElncRNAs and DEGs was calculated by using a statistical method—Pearson correlation. The significant interaction pairs with PPC (Pearson correlation coefficient) > 0.98 and *p* value < 0.05 were selected. The functional enrichment analysis using clusterProfiler was performed to investigate the function of DElncRNAs within the selected significant interaction pairs. The function terms with *p* value < 0.05 were regarded as significant function terms.

### 2.8. Enrichment Map Analysis

The enrichment map analysis using Cytoscape plugin was performed for functional enrichment visualization. The enrichment map organizes enriched terms into a network with edges connecting overlapping gene sets [23]. This map in the present study was constructed to show the similarity among the function terms of genes regulated by lncRNAs. The enriched functions of genes targeted by DElncRNAs can interact with each other instead of being separate and isolated; thus, the dysregulation of a certain function term may result in the aberrant regulation of its interacted functions terms. In the enrichment map, nodes represent the functional GO terms. The color intensity of nodes represents significance (*p* value), and the color of nodes is lighter when the *p* value is bigger. The edge thickness represents the degree of gene overlap that exists between two GO terms. The edge is wider when the mutual gene overlap between two GO terms is larger; that is to say, the similarity between these two GO terms is bigger.

### 2.9. Construction of a DElncRNA-DEG Regulatory Network

The interaction pairs of DElncRNA-DEG were obtained and used to construct a DElncRNA-DEG regulatory network. The topological characteristics of nodes in this network were calculated, and the top 20 nodes were ranked in descending order according to the degree.

### 2.10. Construction of a Transcription Factor- (TF-) DEG Network

First, DEGs obtained from two datasets (GSE92681 and GSE77459) were combined and used for subsequent analysis. The transcription factor- (TF-) DEG interaction pairs were then obtained from several databases, including TRANSFAC [24], TRED [25], and ORTI [26]. Based on these interaction pairs, the TF-DEG regulatory network was constructed. The topological feature of the nodes in this TF-DEG network was calculated, and the top 20 nodes were ranked in descending order according to the degree.

### 2.11. Construction of a ceRNA Network

The miRNAs that target DEGs and miRNAs targeted by DElncRNAs were obtained from the starBase database [17]. Afterward, we integrated coexpressed DElncRNA-mRNA interaction pairs, DElncRNA-miRNA interaction pairs, and DEG-miRNA interaction pairs. Based on these interaction pairs, a ceRNA network was constructed consisting of DElncRNA-miRNA-DEmRNA interaction pairs. The topological feature of nodes in this ceRNA network was calculated, and the top 20 nodes were listed in a descending rank according to the degree. In addition, in order to obtain the functional modules between lncRNA-mRNA interactions and miRNA-mRNA interactions, the Cytoscape plugin MCODE is used to identify the clusters in the ceRNA network.

## 3. Results

### 3.1. The Study Flowchart

**Figure 1:**
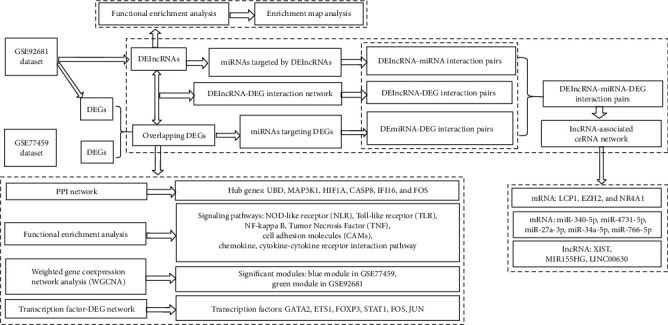
The flowchart of the present study. Two datasets (GSE92681 and GSE77459) were analyzed in this research by using varying bioinformatic analyzing methods, for example, differential expression analysis, functional enrichment analysis, weighted gene coexpression network analysis (WGCNA), enrichment map analysis, and network construction analysis (e.g., TF-DEG network, DElncRNA-DEG network, and ceRNA network).

The analyzing sequence of the present study is presented in Figure 1. As shown in Figure 1, two datasets regarding irreversible pulpitis were analyzed by performing differential expression analysis to identify DEGs and DElncRNAs, by carrying out functional enrichment analysis to identify signaling pathways, by constructing the DEG-TF network to identify critical TFs, and finally by building lncRNA-associated ceRNA network to identify the critical genes, miRNAs, and lncRNAs.

### 3.2. Identification of DEGs

**Figure 2:**
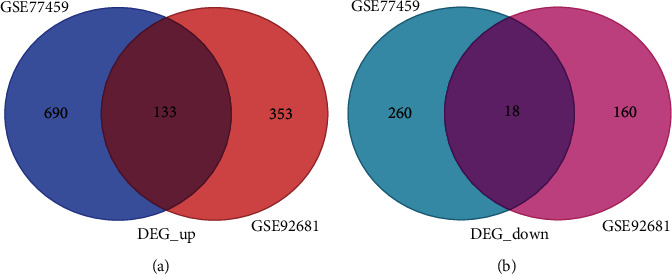
The Venn diagram shows the (a) up- and (b) downregulated DEGs identified by two datasets (GSE77459 and GSE92681). 133 upregulated DEGs and 18 downregulated DEGs were found to be overlapped between DEGs of GSE77459 and GSE92681.

The GSE92681 dataset based on the GPL16956 platform analyzed the noncoding RNA expression profiling data of 7 inflamed pulpal tissues and 5 healthy pulpal tissues (Table 1). The GSE77459 dataset based on the GPL17692 platform analyzed the mRNA expression profiling data of 6 inflamed pulpal tissues and 6 healthy pulpal tissues (Table 1). From the dataset GSE92681, 274 DElncRNAs (138 upregulated and 136 downregulated) and 664 DEGs (486 upregulated and 178 downregulated) were identified (Table 2). From the dataset GSE77459, a total of 1,101 DEGs consisting of 823 upregulated and 278 downregulated were identified (Table 2). In addition, the intersection parts shown in the Venn diagram (Figure 2) show that 151 DEGs including 133 upregulated DEGs and 18 downregulated DEGs were found to be overlapped by two datasets. When considering the union parts of the Venn diagram, a total of 1,176 upregulated DEGs and 438 downregulated DEGs were identified.

### 3.3. Biological Processes and Signaling Pathways Enriched by DEGs

**Figure 3:**
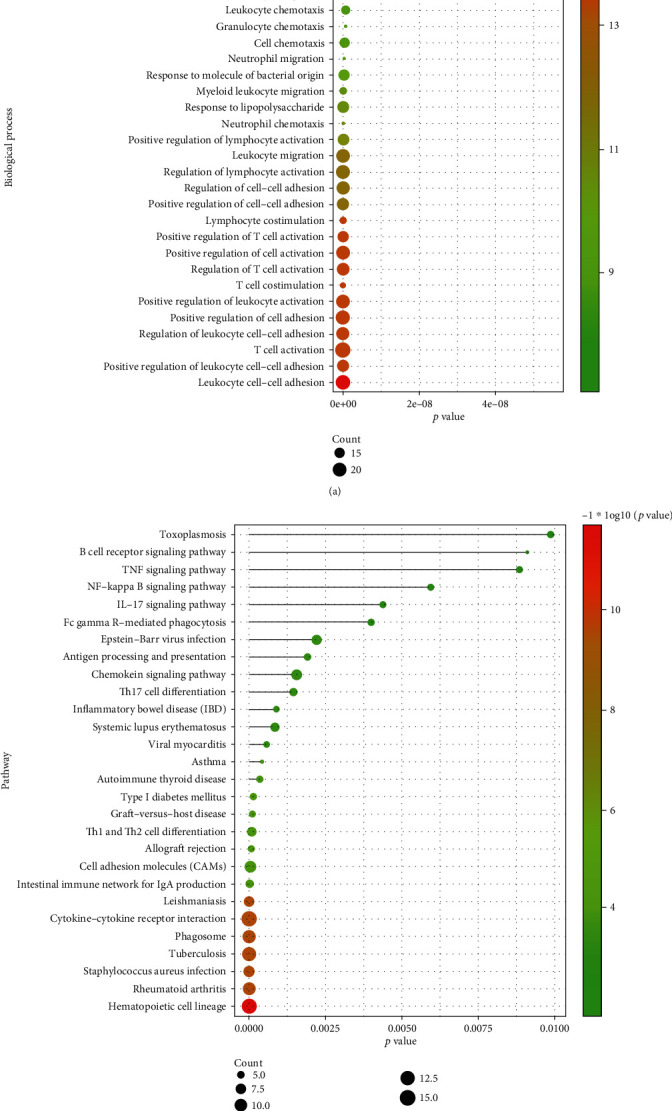
The functional enrichment analysis of DEGs overlapped in two datasets (GSE77459 and GSE92681). (a) The significantly enriched biological processes of overlapped DEGs. (b) The significantly enriched signaling pathways of overlapped DEGs.

As shown in Figure 3(a), DEGs were significantly involved in many biological processes, for instance, cell adhesion-related BPs, immune cells (e.g., 3T cell, neutrophil, granulocyte, leukocyte, and lymphocyte)-related BPs, and immune response-related BPs. As shown in Figure 3(b), DEGs were significantly involved in many signaling pathways, for instance, chemokine and cytokine-related pathways (i.e., TNF, IL-17, chemokine, and cytokine-cytokine receptor interaction), T cell and B cell-related pathways (i.e., B cell receptor and Th1 and Th2 cell differentiation), NF-kappa B, and microbial infection-related pathways (i.e., Epstein-Barr virus infection and *Staphylococcus aureus* infection).

### 3.4. Identification of Hub Genes by Constructing the PPI Network

**Figure 4:**
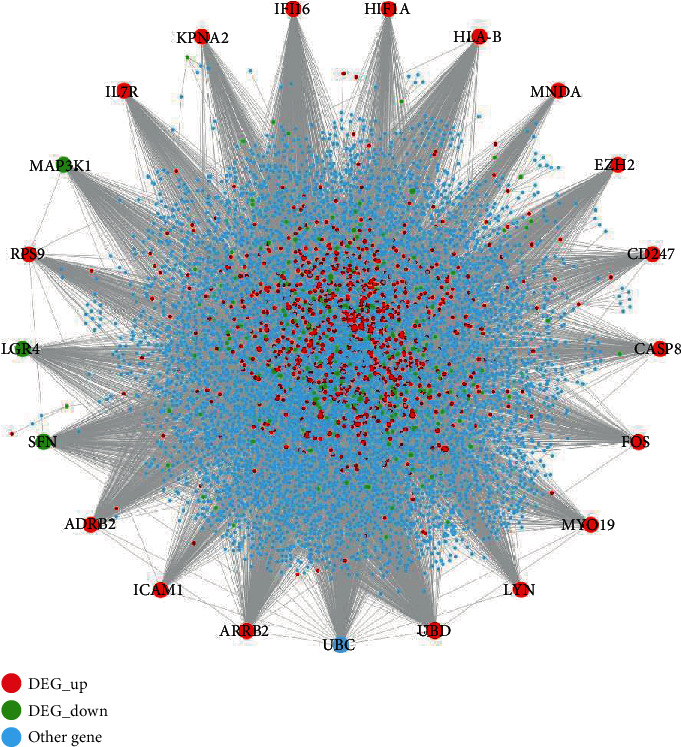
The PPI network of DEGs. The red and green circle nodes represent up- and downregulated DEGs, respectively. The sky-blue circle nodes represent the non-DEGs which interact with DEGs in the PPI network.

The PPI network of DEGs expressed in pulpitis shown in Figure 2 consisted of 9,070 gene nodes and 24,903 PPI interaction pairs. File S1 shows the topological characteristics of all DEG nodes in Figure 2.  Table 3 shows the top 20 gene nodes were ranked in descending order according to their degree. It can be seen from Figure 4 that the gene UBD with the highest degree was identified to play the most important role in the network by interacting with the highest number of DEGs. Apart from the gene UBD, some other upregulated DEGs (e.g., IFI16, ARRB2, HLA-B, EZH2, ADRB2, LYN, FOS, RPS9, KPNA2, IL7R, CASP8, CD247, HIF1A, MYO19, and MNDA) and downregulated DEGs (e.g., SFN, MAP3K1, and LGR4) were also identified to play critical roles in the network.

### 3.5. Enriched Biological Processes of DElncRNAs

**Figure 5:**
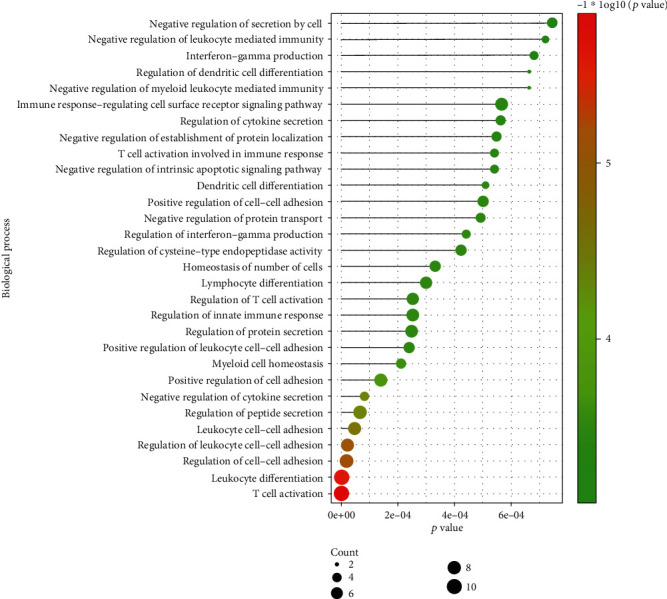
The significantly enriched biological processes of DElncRNAs. Count represents the number of genes enriched in a BP term, and −log10[*p* value] represents the enrichment score. The bigger size of the dots corresponding to a BP term means more genes were enriched in this term. The colored dots represent the term enrichment: green indicates low enrichment, and red indicates high enrichment.

As shown in Figure 5, DElncRNAs were found to be significantly involved in many biological processes, for example, immune cells (dendritic cells, leukocytes, and T cells)-related BPs (e.g., regulation of dendritic cell differentiation, dendritic cell differentiation, T cell activation involved in immune response, regulation of T cell activation, and leukocyte differentiation), cytokine-related BPs (i.e., interferon-gamma production, regulation of cytokine secretion, and negative regulation of cytokine secretion).

### 3.6. The Similarity of Functional Terms of DElncRNAs

**Figure 6:**
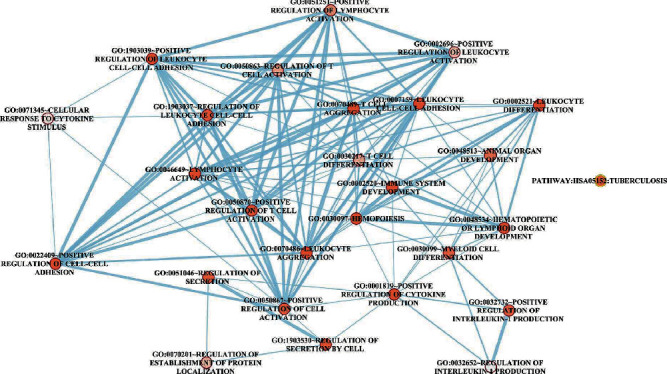
The enrichment map shows the GO interacting network of genes targeted by DElncRNAs. The orange circle node represents the significantly enriched GO terms, and the line represents the interaction between GO terms.

As shown in Figure 6, immune cells (e.g., lymphocytes, leukocytes, and T cells)-related GO functional terms were observed to interact with cytokine-related GO terms (e.g., the cellular response of cytokine stimulus, positive regulation of cytokine production, and regulation of interleukin-1 production).

### 3.7. The DElncRNA-DEG Regulatory Network

**Figure 7:**
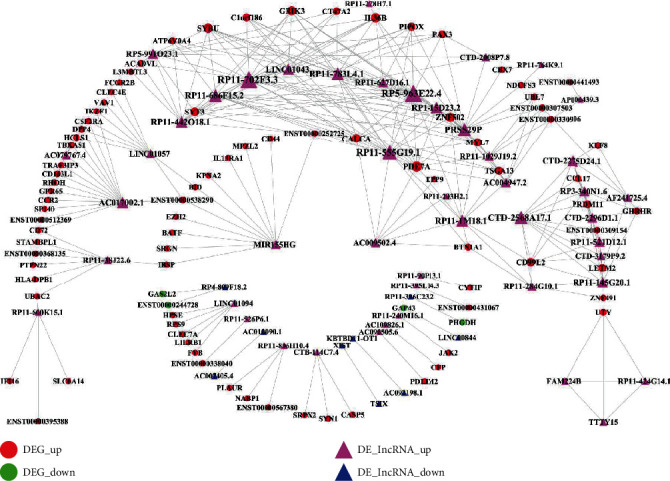
The DElncRNA-DEG regulatory network involved in pulpitis. The red circle nodes represent the upregulated DEGs, and the green circle nodes represent the downregulated DEGs. The rose-red triangle nodes represent the upregulated DElncRNA, and the blue triangle node represents the downregulated DElncRNA.

As shown in Figure 7, the DElncRNA-DEG regulatory network consisted of 312 nodes and 905 edges. File S2 shows the characteristics of all nodes in the network in Figure 7. As seen from Table 4, many lncRNAs with the highest degree play critical roles in the network, such as RP11-702F3.3, RP5-963E22.4, RP11-555G19.1, CTD-2568A17.1, and PRSS29P.

### 3.8. Identification of Hub Transcription Factor

**Figure 8:**
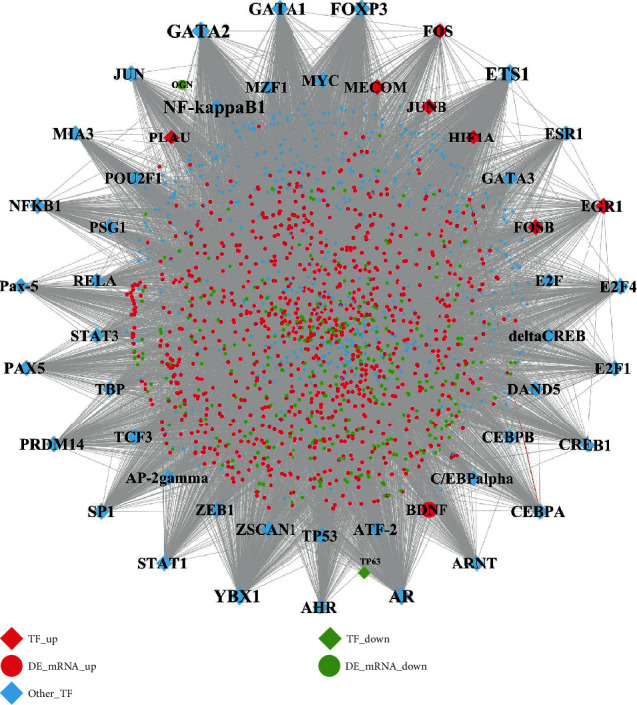
The TF-DEG regulatory network involved in pulpitis. The diamond nodes represent TFs and the circle nodes represent DEGs. For the diamond nodes, the red diamond nodes represent the upregulated TFs, the green diamond nodes represent the downregulated TFs, and the sky-blue diamond nodes represent other nondifferentially expressed TFs. For the circle nodes, red circle nodes represent the upregulated DEGs, while the green circle nodes represent the downregulated DEGs.

The TF-DEG regulatory network consisted of 1,750 nodes and 17,095 edges (Figure 8). File S3 shows the topological characteristics of all nodes in the network in Figure 8. Combining the data shown in Figure 8 and Table 5, it can be found that only one TF-FOS was differentially expressed in pulpitis among the top 20 nodes of the TF-DEG network. Although the other 19 nodes (i.e., GATA2, ETS1,YBX1, AR, FOXP3, GATA1, SP1, E2F4, PRDM14, ARNT, MIA3, JUN, CREB1, FOS, STAT1, CEBPA, AHR, E2F1, PAX5, and Pax-5) except FOS were not differentially expressed in pulpitis, they still play critical roles in the pathogenesis of pulpitis by interacting and regulating DEGs that are expressed in pulpitis. As seen from Figure 8, some other TFs were also found to be differentially expressed in pulpitis, for example, some FOSB, JUNB, EGR1, HIF1A, PLAU, MECOM, TP63, and BDNF.

### 3.9. Identification of Significant Gene Modules

**Figure 9:**
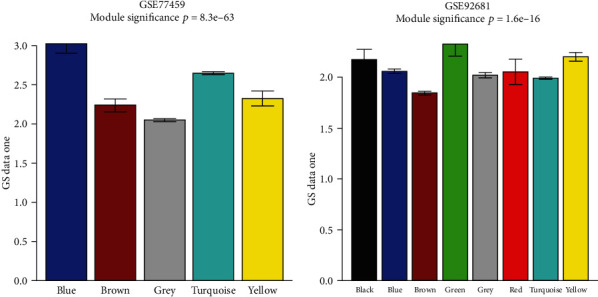
The coexpressed gene modules identified in GSE77459 and GSE92681. The horizontal axis represents each different color module; the vertical axis represents the correlation coefficient between genes in each module and disease status.

As seen from Figure 9, five gene modules with varying colors (i.e., blue, brown, grey, turquoise, and yellow) were identified from GSE77459. Among these five coexpressed gene modules, the blue module with the lowest *p* value was found to be the most significant module. Regarding GSE92681, eight gene modules with various colors (i.e., black, blue, brown, green, grey, red, turquoise, and yellow) were identified, among which the green module with the lowest *p* value was found to be the most significant module.

### 3.10. Construction of PPI Network for Selected Significant Gene Modules

**Figure 10:**
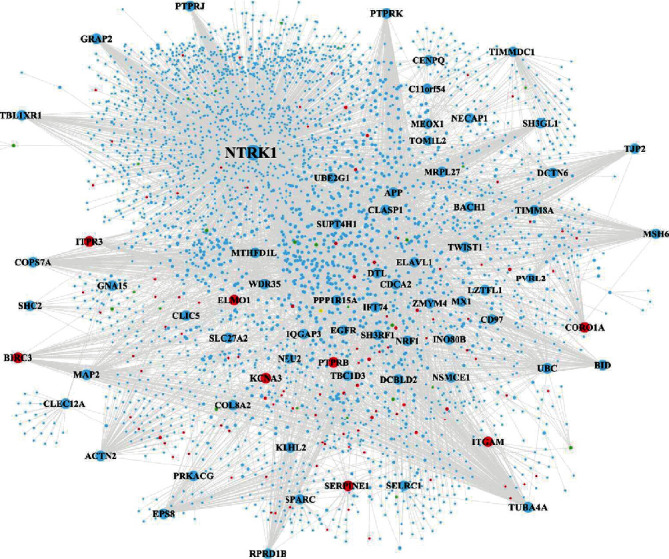
The PPI network of significant modules within the blue module in GSE77459. The red and green circle nodes represent up- and downregulated DEGs, respectively. The sky-blue circle nodes represent the non-DEGs which interact with DEGs in the PPI network.

**Figure 11:**
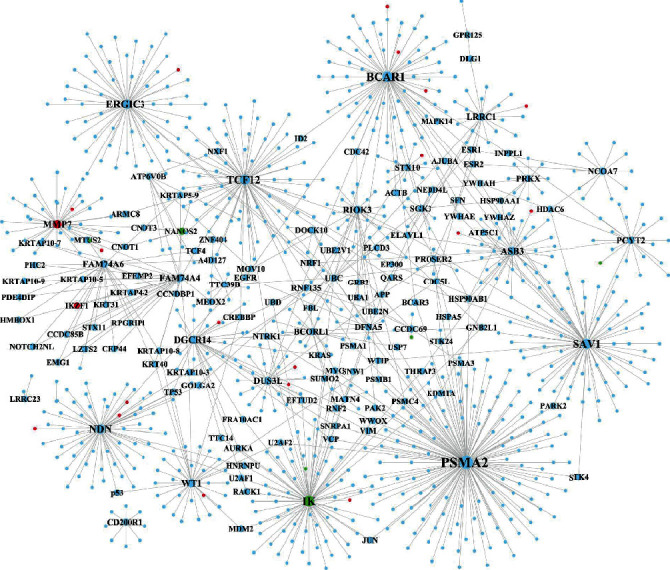
The PPI network of significant modules within the green module in GSE92681. The red and green circle nodes represent up- and downregulated DEGs, respectively. The sky-blue circle nodes represent the non-DEGs which interact with DEGs in the PPI network.

The PPI networks were constructed for these two selected significant coexpressed gene modules (blue module in GSE77459 (Figure 10) and green module in GSE92681 (Figure 11)), respectively. The PPI network of the blue module within the GSE77459 dataset consisted of 3,599 gene nodes and 4,712 edges, while the PPI network of the green module within the GSE92681 dataset consisted of 930 gene nodes and 1,019 edges. Files S4 and S5 show the topological characteristics of all nodes of the network shown in Figures 10 and 11, respectively. Tables 6 and 7 show the topological characteristics of these two PPI networks depicted in Figures 10 and 11, respectively. Among the top 25 genes in the PPI network of the blue module of the GSE77459 dataset, only 3 upregulated DEGs (BIRC3, ITPR3, and PTPRB) were found; by contrast, the other 22 genes within the top 25 gene nodes were not DEGs (Table 6). Among the top 25 genes in the PPI network of the green module of the GSE92681 dataset, only one upregulated DEG (MMP-7) and one downregulated DEG (IK) were found; by contrast, the other 23 genes were not DEGs (Table 7).

### 3.11. The ceRNA Network

**Figure 12:**
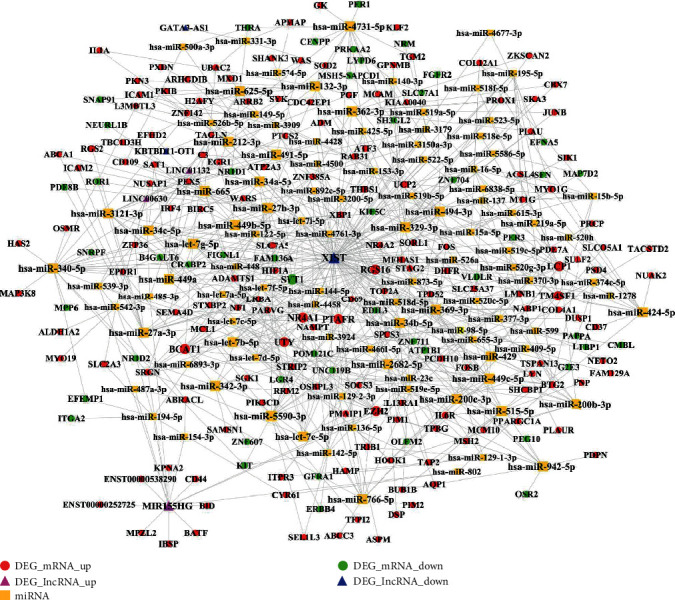
The ceRNA network consisting of DElncRNA-miRNA-DEG interaction pairs. The red circle nodes represent the upregulated DEGs, and the green circle nodes represent the downregulated DEGs. The yellow square nodes represent miRNA. The rose-red triangle nodes represent the upregulated DElncRNA, and the blue triangle node represents the downregulated DElncRNA.

**Figure 13:**
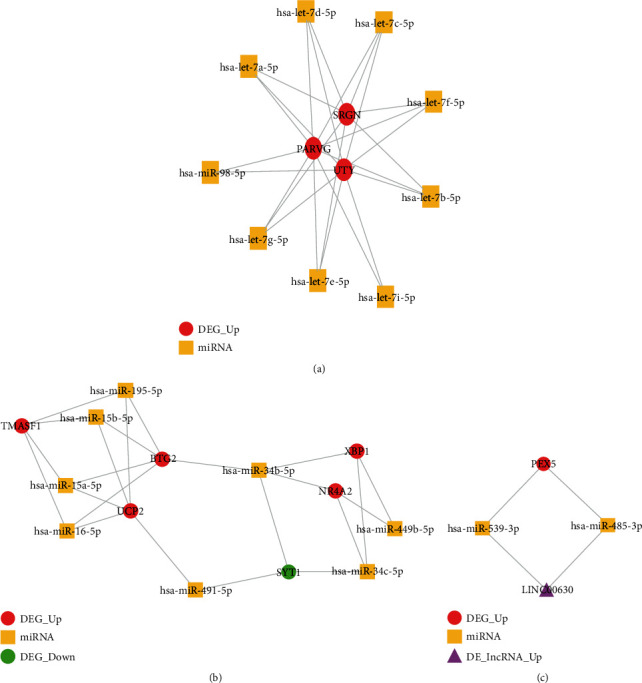
Three clusters identified in the ceRNA network. The red circle nodes represent the upregulated DEGs, and the green circle nodes represent the downregulated DEGs. The yellow square nodes represent miRNA. The rose-red triangle nodes represent the upregulated DElncRNA.

As shown in Figure 12, a ceRNA network consisting of DElncRNA-miRNA-DEmRNA interaction pairs was depicted. File S6 shows the topological characteristics of all nodes in the network in Figure 12. Combined with the information of the top 20 nodes shown in Table 8, it can be observed that lncRNA XIST plays the most important role in this network. Apart from lncRNA XIST, some genes (e.g., MIR155HG, LCP1, EZH2, and NR4A1) and several miRNAs (e.g., hsa-miR-340-5p, hsa-miR-4731-5p, hsa-miR-5590-3p, hsa-miR-27a-3p, hsa-miR-27b-3p, hsa-miR-329-3p, hsa-miR-362-3p, hsa-miR-494-3p, hsa-miR-424-5p, hsa-miR-2682-5p, hsa-miR-515-5p, hsa-miR-766-5p, hsa-miR-449c-5p, hsa-miR-34a-5p, and hsa-miR-449a) also play critical roles in the network. As shown in Figures 13(a)–13(c), three clusters were identified from the ceRNA network. As seen from Figure 13(c), LINC00630 can compete with two miRNAs (miR-539-3p and miR-485-3p) in targeting gene PEX5, and LINC00630 can indirectly target gene PEX5.

## 4. Discussion

This study identified many genetic and epigenetic biomarkers involved in the pathology of pulpitis, including six hub genes in the PPI network (i.e., UBD, MAP3K1, HIF1A, CASP8, IFI16, and FOS), several factors involved in the ceRNA network (e.g., three genes (i.e., LCP1, EZH2, and NR4A1), five miRNAs (i.e., miR-340-5p, miR-4731-5p, miR-27a-3p, miR-34a-5p, and miR-766-5p), and three lncRNAs (i.e., XIST, MIR155HG, and LINC00630)), six transcription factors (i.e., GATA2, ETS1, FOXP3, STAT1, FOS, and JUN), and seven signaling pathways (i.e., NOD-like receptor (NLR), Toll-like receptor (TLR), NF-kappa B, tumor necrosis factor (TNF), cell adhesion molecules (CAMs), chemokine, and cytokine-cytokine receptor interaction pathway). The detailed roles of these critical factors are supported by the previous scholar evidence and will be described in the following section.

Many genes are identified to be involved in the PPI network of pulpitis; however, there is still no direct evidence that can support the involvement of these genes in pulpitis. Herein, only six genes that were most investigated by previous research were described, including UBD, MAP3K1, HIF1A, CASP8, IFI16, and FOS. For the first example, UBD (Ubiquitin D) was shown to have multiple cellular processes that occurred in pulpitis: regulating NF-kappa B signaling pathway [27], mediating cell apoptosis in a caspase-dependent manner [28], and being involved in the maturation of dendritic cells [29]. Looking at the case of MAP3K1 (Mitogen-Activated Protein Kinase Kinase Kinase 1), this gene encodes a serine/threonine kinase and has been shown to be part of many signaling transduction cascades including ERK (extracellular signal-regulated kinases) [30] and JNK (c-Jun N-terminal kinase) kinase [31], NF-kappa B [32], TLR4 signaling [33], and IL-1 family signaling pathways [32]. Since these pathways mentioned here have been verified to be implicated in pulpitis [34–36], MAP3K1 can be speculated to be also involved in pulpal inflammation. Taking the case of HIF1A (hypoxia-inducible factor 1 subunit alpha), this gene encodes the alpha subunit of transcription factor hypoxia-inducible factor-1 (HIF-1) [37]. This gene has been shown to regulate the cellular and systemic homeostatic response to the hypoxia environment by activating many genes related to angiogenesis and apoptosis [38]. Since the hypoxic environment caused by a collapse of the venous microcirculation during the pulpal inflammation could result in localized or generalized pulp necrosis and death [39], HIF1A could be a specific signal which indicates the potential deterioration risk from irreversible pulpitis to pulp necrosis and death. For example, caspase-8 and caspase-9 (encoded by CASP8 and CASP9) are cysteine proteases that play a crucial role in the signaling pathways of apoptosis, necrosis, and inflammation [40]. Since CASP9 is involved in cell apoptosis in human dental pulp stem cells from deciduous teeth [41] and also activation of caspase-9 can lead to activation of downstream caspase-8 [42], CASP8 can be therefore assumed to be involved in the signaling pathway of apoptosis in the pathogenesis of pulpitis. Another example is interferon gamma inducible protein 16 (IFI16) that is induced by IFN-*γ*, a member of the HIN-200 family of cytokines. A high prevalence of IFN-*γ* messenger RNA in inflamed pulps has been detected [43], and the methylated status of IFN-*γ* has been altered from total methylation in healthy pulp to partial methylation or unmethylation in the inflamed pulp. Since IFN-*γ* cytokine may be implicated in the immune response during the process of pulp inflammation [44], the epigenetic events of pulpitis could also be relevant to the alteration of IFI16. Looking at another example, the Fos gene family (FOS, FOSB, FOSL1, and FOSL2) has been suggested to regulate the process of cell proliferation, differentiation, transformation, and apoptosis. The expression of the immediate-early gene product Fos was reported to be evoked by the LPS-induced pulpal inflammation in the rostral trigeminal regions of ferrets [45]. Regarding the pattern of its expression, another study using the rat model found that the expression of Fos induced by chronic tooth pulpal inflammation in dynorphin-rich regions of rat brainstem was shown to be temporal and spatial [46]. The role of almost all of the genes in pulpitis is based on speculation and thus needs to be validated in future research by designing relevant experiments.

Three genes (i.e., LCP1, EZH2, and NR4A1) are identified to be key biomarkers in the ceRNA network. For example, LCP1 (Lymphocyte Cytosolic Protein 1) is significantly enriched in a GO term named T cell activation [47]. The activation of T lymphocytes can orchestrate other types of immunocompetent cells, thereby promoting the local immune defense that occurred in the dental pulp [48]. Given this evidence, it can be assumed that LCP1 might be involved in the pathogenic mechanism of pulpitis by regulating T cell-mediated immune response. For another example, the enhancer of zeste homolog 2 (EZH2), as a catalytic subunit of PRC2 (polycomb repressor complex 2), could regulate gene silencing via its histone methyltransferase activity, accumulation of DNA damage, and chromosome abnormalities [49]. EZH2 is suggested to be implicated in the pulp inflammation, proliferation, and regeneration by inhibiting osteogenic differentiation of human dental pulp cell (HDPCs) and enhancing inflammatory response and proliferation [50]. Another research investigating the effect of EZH2 in odontogenic differentiation of hDPCs suggested that EZH2 could impair mineralization of HDPCs under the mechanism of activating the Wnt canonical signaling pathway [51]. Taking the final example, Nuclear Receptor Subfamily 4 Group A Member 1 (NR4A1) is enriched in many pulpitis-related signaling pathways, including signaling by PDGF and EGFR, PI3K/AKT activation, and MAPK signaling pathways, and also some GO terms including positive regulation of endothelial cell proliferation and apoptotic process. The previous scholar evidence investigating the involvement of NR4A1 in inflammation showed that the overexpression of NR4A1 was associated with a chronic low-grade inflammatory state [52] and also plays a key role in mediating the anti-inflammatory effects of apoptotic cells [53]. However, the expression patterns and its regulatory mechanisms of NR4A1 remain to be researched in pulp inflammation.

Many miRNAs are involved in the ceRNA network of pulpitis; however, the expression patterns and functions of almost all of them have not been investigated in pulpal inflammation. Based on the potential target genes of the miRNAs searched on the miRWalk database [54], some miRNAs (i.e., miR-340-5p, miR-4731-5p, miR-27a-3p, miR-34a-5p, and miR-766-5p) could be assumed to be implicated in pulpitis by targeting genes related to inflammatory response and regeneration. For the first example, miR-340-5p has been validated to target gene LIMS1 (LIM Zinc Finger Domain Containing 1), the encoded protein of which is involved in the integrin signaling [55]. Sine integrin-associated signaling is implicated in the odontogenic stimulation of human dental pulp stem cells [56]; miR-340-5p might be involved in the pulp healing and regeneration during the pathogenic processes of pulpitis. In the case of miR-4731-5p, it has been validated to target gene IRAK4 (Interleukin 1 Receptor-Associated Kinase 4), which encodes a kinase that can activate the upregulation of NF-kappa B [57]. Since NF-kappa B has been found to be activated by lipopolysaccharide (LPS) and tumor necrosis factor (TNF) in the dental pulp stem cells (DPSCs) and further implicated in the immune response of pulpal infection [35], miR-4731-5p could be regarded as an inflammatory biomarker during the pulpal inflammation. In the case of miR-27a-3p, it has been found to regulate the cell proliferation of vascular endothelial cells positively and further being implicated in the angiogenesis and neovascularization through ERK1 and ERK2 cascade [58]. Since an increased number of blood vessels have been found in the inflamed human dental pulp [59], miR-27a-3p could be involved in the pulpal regeneration by mediating angiogenesis during the process of pulpitis. Taking the example of miR-34a-5p, it has been validated to target the gene MAP2K1 (Mitogen-Activated Protein Kinase Kinase 1), which encodes a dual-specificity kinase that has been well-known to be involved in the ERK pathway [60]. Since the MAP/ERK pathway is implicated in the differentiation and stimulation of odontoblasts during reactionary dentinogenesis [61], miR-34a-5p might be involved in the dentinogenesis-based repair mechanism during the pathogenesis of pulpitis. In the case of miR-766-5p, it is one of the subtypes of miR-766 which is upregulated in inflamed pulpitis compared to the normal pulps [62]. miR-766 can target Heat Shock Transcription Factor 1 (HSF1), which encodes a transcription factor that can be rapidly induced after temperature stress [63]. Since thermal stresses, including hot and cold loadings, may induce the activation of tooth pain signaling [64], miR-766 could be assumed to be a sensitive biomarker of thermal exposure.

Three lncRNAs (i.e., XIST, MIR155HG, and LINC00630) are identified to be key factors involved in the ceRNA network of pulpitis. For the first example, the lncRNA X Inactive Specific Transcript (XIST), as a 17 kb long RNA transcribed by the inactive X chromosome, is involved in the X chromosome inactivation in female mammals, thus providing dosage equivalence between males and females [65]. More and more scholarly evidence has shown that XIST is dysregulated in many cancers and inflammatory conditions [66–69]. A recent study found that XIST can mediate the inflammation process of mammary epithelial cells by regulating the NF-*κ*B/NLRP3 inflammasome pathway [70]. In the case of the lncRNA MIR155HG (MIR155 Host Gene), it is formerly known as BIC (B-cell integration cluster) and has been shown to function as a primary micro (mi)RNA for miR-155 [71]. Since miR-155 has been established to be an ancient master regulator of the immune response [72], the MIR155HG/miR-155 axis may be involved in many physiological and pathological processes including inflammation and immunity [73]. In the case of LINC00630, this lncRNA can interact with miR-539-3p, miR-485-3p, and PEX5 gene and combinedly generate a closed regulatory loop in the ceRNA network. The gene PEX5 (Peroxisomal Biogenesis Factor 5) encodes the type 1 peroxisomal targeting signal (PTS1) receptor, which is one of 15 peroxins required for peroxisome biogenesis [74]. A recent study showed that peroxisomes could resolve microbial infection by modulating many innate immune-related pathways (reactive oxygen species (ROS) and reactive nitrogen species (RNS) signaling) and activating the stress response kinase p38 [75]. Based on the finding of the PEX5/LINC00630/miR-539-3p/miR-485-3p loop, this loop may be required for promoting the immune response in pulpal inflammation.

Several transcription factors have been identified to be involved in the TF-gene regulatory network of pulpitis, including GATA2, ETS1, FOXP3, STAT1, FOS, and JUN. GATA2 (Endothelial Transcription Factor GATA-2) is a transcriptional activator that regulates the expression of the endothelin-1 gene in endothelial cells [76]. It has been shown that endothelial cells can influence DPSCs by secreting endothelin-1 and further promoting the odontogenic differentiation of DPSCs [77]; thus, GATA2 can be assumed to be involved in the restoration and regeneration of dental pulp. Taking the example of ETS1, it could be speculated to be involved in the inflammation and regeneration of pulp based on its dual functions: controlling the expression of many cytokines as well as chemokine genes [78], being implicated in angiogenesis by regulating the expression of genes that are associated with migration and invasion of endothelial cells [79]. Taking the case of FOXP3 (Forkhead Box P3), it is the most specific biomarker of regulatory T cells (Treg) [80, 81]. Treg as a subset of T lymphocytes has been playing a pivotal role in the immune and inflammatory response of pulpitis by secreting anti-inflammatory cytokines, including interleukin-10 and transforming growth factor b (TGF-b) [82]. Based on this, FOXP3 can be speculated to be involved in the inflammatory response by regulating the cytokine genes. In the case of STAT1 (Signal Transducer And Activator Of Transcription 1), it has been shown to play a critical role in mediating the cellular responses to many inflammatory mediators involved in pulpitis, including interferons (IFNs), cytokines (IL1, IL6, and KITLG/SCF), and growth factors (epidermal growth factors (EGF) and platelet-derived growth factor (PDGF)) [83]. For example, IFN-gamma has been shown to be a feasible modulator to improve the dentinogenic and immunosuppressive functions of irreversible pulpitis-DPSCs [84]; cytokines as a crucial part of host response could be regarded as diagnostic markers of pulpal inflammation [85, 86]; and growth factors can contribute to the angiogenic response of pulp tissue and enhance the regeneration of pupal-like tissue [87, 88]. Taking the final example, AP-1 complex consisting of c-JUN and c-FOS can synergize with Smad3/Smad4 protein and further cooperatively mediate the transforming growth factor-beta (TGF-beta) signaling pathway [89]. Since TGF-beta has been well known to stimulate odontoblast cells to secrete reactionary dentin [90], JUN and FOS can be speculated to play a role in the repair and regeneration process of the dental pulp.

Seven signaling pathways have been identified to be significantly enriched in the pathogenesis of pulpitis, for example, NOD-like receptor (NLR), Toll-like receptor (TLR), NF-kappa B, tumor necrosis factor (TNF), cell adhesion molecules (CAMs), chemokine, and cytokine-cytokine receptor interaction pathways. All of the pathways listed above have been well supported by previous studies. In the first example, the nucleotide-binding oligomerization domain- (NOD-) like receptors (NLRs) and Toll-like receptors (TLRs) are two members of the pattern recognition receptor (PRR) family. It has been shown/demonstrated by authors that PRR family members can recognize caries pathogen-associated molecular patterns (PAMPs) and play crucial roles in the initiation of dental pulp innate immunity [91]. In another example, the downregulation of the NF-kappa B gene was suggested to enhance the odontogenic differentiation of DPSCs and the formation of the collagen matrix, indicating that NF-kappa B could be a potential target for promoting pulp tissue regeneration [92]. Taking the tumor necrosis factor-*α* (TNF-*α*) as an example, it has been shown that TNF-*α* is a pleiotropic cytokine that is upregulated in pulpal tissues of teeth with irreversible pulpitis [93]. Not only does TNF-*α* play a role in promoting inflammation by recruiting leukocytes and stimulating the production of proinflammatory cytokines, but it may also cause pain hypersensitivity by directly acting on nociceptive neurons [94]. In the case of cell adhesion molecules (CAMs), diverse CAM molecules (e.g., platelet-endothelial cell adhesion molecule-1 (PECAM-1), intercellular adhesion molecule-1 (ICAM-1), intercellular adhesion molecule-3 (ICAM-3), and vascular cell adhesion molecule-1 (VCAM-1)) were shown to be expressed in the vascular endothelium of the inflamed human dental pulp, by playing roles in promoting transendothelial migration of leukocytes from the bloodstream into tissue [95]. Finally, chemokines and cytokines are kinds of inflammatory mediators suggested being involved in the innate immune response of pulpitis, playing protective roles in attracting varying inflammatory cells, inducing antibacterial reactions by the production of antimicrobial peptides such as defensins, and further killing cariogenic microbial [96]. All of the signaling pathways listed above can form complicated interactions and are involved in the inflammatory immune response of pulpitis. However, it is also worthwhile to note that the pathways identified in this study have also been documented as the classic pathways involved in all inflammatory diseases and are not specific for pulpitis. It is therefore questionable to regard these pathways as therapeutic targets that can inhibit the progression of pulpitis.

Some limitations should be acknowledged in this study. First, only expression profiling datasets of lncRNAs and genes could be obtained, and there were no miRNA expression profile datasets related to pulpitis in the GEO dataset. It was therefore impossible to predict the expression tendency of miRNAs in the pathology of pulpitis. This also means that miRNA sequencing technology needs to be applied to investigate the alteration of miRNAs in pulpitis. Second, the sample size of the datasets included was small (GSE92681: 12; GSE77459: 12), and the analysis based on this limited sample data may result in a decrease of the prediction accuracy. Third, it should be noted that only bioinformatic techniques were employed. And because of limited funding, no clinical experiments were performed to validate the expression of the RNA molecules predicted in pulpitis. Although this study has some limitations, the findings also provide some direction for future research. First, the biomarkers identified could be promising therapeutic targets that can lay the groundwork for future experimental research design. Second, the identification of these biomarkers can benefit the research of pulp tissue engineering, based on the evidence that genetically modified stem cells will receive better treatment efficacy compared with stem cells alone. The combined application of these genetic and epigenetic biomarkers modified DPSCs and already validated biomaterial scaffold (e.g., collagen, poly (lactic) acid, and fibrin) is promising for future regenerative endodontic therapy.

## Figures and Tables

**Table 1:** The pulpitis-related datasets used for the present analysis.

Datasets	Experimental type of datasets	Sample size of inflamed pulp tissue	Sample size of healthy pulp tissue
GSE92681	Noncoding RNA	7	5
GSE77459	mRNA	6	6

**Table 2:** The number of upregulated and downregulated DEGs or DElncRNAs identified from included datasets.

Datasets	Number of upregulated factors	Number of downregulated factors	Number of total factors
GSE92681_DElncRNA	138	136	274
GSE92681_DEG	486	178	664
GSE77459_DEG	823	278	1101

**Table 3:** The topological characteristics of the top 20 nodes in the PPI network.

Gene name	Degree	Average shortest path length	Betweenness centrality	Closeness centrality	Clustering coefficient	Topological coefficient
UBD	656	2.700089	0.092136	0.370358	0.001564	0.003702
UBC	460	2.20363	0.309699	0.453797	0.004092	0.005551
IFI16	436	2.853032	0.052658	0.350504	0.001381	0.005531
ARRB2	369	2.872067	0.040035	0.348181	0.001915	0.007365
HLA-B	338	2.825255	0.037735	0.35395	0.002915	0.00551
EZH2	301	2.891545	0.030774	0.345836	0.00206	0.008083
SFN	296	2.903608	0.032059	0.344399	0.00213	0.00725
ADRB2	294	2.855246	0.033685	0.350233	0.003088	0.006755
LYN	243	2.795595	0.03365	0.357706	0.013332	0.008931
MAP3K1	229	2.901173	0.021399	0.344688	0.00406	0.009621
FOS	226	2.943448	0.02579	0.339738	0.00468	0.010265
RPS9	208	2.82127	0.017896	0.35445	0.009337	0.00958
KPNA2	205	2.867641	0.022731	0.348719	0.00263	0.009771
IL7R	199	2.931496	0.015151	0.341123	0.002944	0.011601
LGR4	198	3.039287	0.020276	0.329025	3.59E-04	0.010662
CASP8	196	2.906707	0.020462	0.344032	0.008791	0.010607
CD247	196	2.964697	0.024808	0.337303	0.00539	0.010402
HIF1A	174	2.950089	0.016343	0.338973	0.003123	0.013813
MYO19	173	2.93548	0.0148	0.34066	0.010687	0.00917
MNDA	168	3.236498	0.007305	0.308976	7.84*E* − 04	0.012766

**Table 4:** The topological characteristics of the top 20 nodes in the DElncRNA-DEG regulatory network.

Name	Degree	Average shortest path length	Betweenness centrality	Closeness centrality	Clustering coefficient	Topological coefficient	Regulate
RP11-702F3.3	32	2.26666667	0.09257337	0.44117647	0.36363636	0.2867215	lncRNAup
RP5-963E22.4	32	2.05	0.14685811	0.48780488	0.3982684	0.27954545	lncRNAup
RP11-555G19.1	25	2.21666667	0.08384754	0.45112782	0.37908497	0.30401235	lncRNAup
CTD-2568A17.1	21	2.56666667	0.17922917	0.38961039	0.37179487	0.26627219	lncRNAup
PRSS29P	21	2.28333333	0.18869008	0.4379562	0.20588235	0.26916221	lncRNAup
RP11-686F15.2	20	2.53333333	0.01950748	0.39473684	0.44761905	0.35757576	lncRNAup
RP11-783L4.1	19	2.58333333	0.01555518	0.38709677	0.47252747	0.37327189	lncRNAup
RP11-1M18.1	18	2.06666667	0.32645541	0.48387097	0.3030303	0.22644928	lncRNAup
LINC01043	17	2.61666667	0.01053104	0.38216561	0.43589744	0.36923077	lncRNAup
RP1-15D23.2	17	2.48333333	0.00914061	0.40268456	0.59090909	0.38333333	lncRNAup
RP11-442O18.1	17	2.66666667	0.03491119	0.375	0.3974359	0.33846154	lncRNAup
AC017002.1	16	2.35	0.51050061	0.42553191	0.05714286	0.17333333	lncRNAup
CTD-2275D24.1	14	3.23333333	0.02043004	0.30927835	0.44444444	0.41176471	lncRNAup
PDE7A	13	2.15	0.08844751	0.46511628	0.35897436	0.33208255	mRNA up
RP11-145G20.1	13	3.33333333	0.01660117	0.3	0.36111111	0.39869281	lncRNAup
RP11-521D12.1	13	3.35	0.00459174	0.29850746	0.5	0.43382353	lncRNAup
RP5-991O23.1	13	3.03333333	0.01404135	0.32967033	0.33333333	0.31481481	lncRNAup
CTD-2296D1.1	12	3.36666667	0.00429011	0.2970297	0.5	0.453125	lncRNAup
RP3-340N1.6	12	3.35	0.0054601	0.29850746	0.57142857	0.44852941	lncRNAup
MIR155HG	11	2.55	0.41865079	0.39215686	0	0.12121212	lncRNAup

**Table 5:** The topological characteristics of the top 20 nodes in the TF-DEG regulatory network.

Name	Degree	Average shortest path length	Betweenness centrality	Closeness centrality	Clustering coefficient	Topological coefficient
GATA2	518	2.03887936	0.11659147	0.49046551	0.00308432	0.02016302
ETS1	508	2.05603202	0.11527216	0.48637375	0.0028654	0.02038007
YBX1	425	2.22012579	0.09284757	0.45042493	0.00137625	0.02440558
AR	414	2.18753573	0.08279112	0.45713539	0.0032635	0.02330064
FOXP3	407	2.24013722	0.09211348	0.44640123	0.00128297	0.02202286
GATA1	322	2.30360206	0.03470622	0.43410276	0.00336681	0.02811219
SP1	214	2.3619211	0.02271018	0.42338417	0.00842438	0.03168479
E2F4	203	2.50714694	0.02510997	0.39885975	0.00248744	0.03155395
PRDM14	194	2.47970269	0.01761185	0.40327415	0.00459377	0.03274385
ARNT	190	2.47970269	0.00990145	0.40327415	0.00668338	0.04482786
MIA3	182	2.51172098	0.00828163	0.39813339	0.00558557	0.04629319
JUN	179	2.45740423	0.0132459	0.40693346	0.01726194	0.03584432
CREB1	178	2.43853631	0.0134572	0.41008206	0.01314035	0.03899076
FOS	178	2.04116638	0.03258878	0.48991597	0.05986161	0.0352397
STAT1	176	2.51229274	0.01672367	0.39804279	0.00525974	0.03983636
CEBPA	168	2.58604917	0.01187163	0.38669025	0.00377816	0.04812159
AHR	168	2.53459119	0.00776619	0.39454094	0.00620188	0.04722287
E2F1	165	2.50085763	0.01123109	0.39986283	0.00657797	0.04104892
PAX5	164	2.51172098	0.00771197	0.39813339	0.00890319	0.0434212
Pax-5	161	2.56089194	0.0066902	0.39048895	0.00535714	0.04834386

**Table 6:** The topological characteristics of the top 25 gene nodes in the PPI network of the blue module within the GSE77549 dataset.

Name	Degree	Betweenness centrality	Closeness centrality	Clustering coefficient	Topological coefficient	Regulate
NTRK1	1981	0.797678	0.550769	8.10*E* − 05	0.00147	Other_gene
TUBA4A	182	0.057422	0.34576	2.46*E* − 04	0.012097	Other_gene
PTPRK	110	0.048111	0.334924	5.10*E* − 04	0.017763	Other_gene
ACTN2	101	0.04028	0.340466	8.08*E* − 04	0.012519	Other_gene
MSH6	98	0.029296	0.414544	0.009211	0.011399	Other_gene
COPS7A	87	0.027065	0.331267	0	0.024951	Other_gene
BIRC3	79	0.030191	0.33259	0	0.022419	mRNA up
SH3GL1	79	0.025169	0.330228	0	0.023044	Other_gene
EPS8	78	0.030772	0.33259	0	0.022436	Other_gene
PTPRJ	76	0.02319	0.408163	0.008288	0.014223	Other_gene
UBC	75	0.16533	0.47619	0.005405	0.017173	Other_gene
RPRD1B	71	0.019601	0.329863	0	0.026232	Other_gene
TJP2	68	0.019477	0.328079	0	0.027715	Other_gene
BID	65	0.018865	0.33035	0	0.030769	Other_gene
TBL1XR1	62	0.023597	0.409283	0.010929	0.017196	Other_gene
GRAP2	59	0.019032	0.330838	6.05*E* − 04	0.027809	Other_gene
MAP2	57	0.016676	0.406541	0.014935	0.018914	Other_gene
TIMMDC1	53	0.022335	0.327539	7.54*E* − 04	0.025227	Other_gene
BACH1	48	0.013952	0.327929	0	0.036859	Other_gene
DTL	48	0.009929	0.332961	0.006475	0.032282	Other_gene
CORO1A	46	0.016515	0.327929	0	0.029927	mRNA up
MX1	43	0.013576	0.329347	0	0.037265	Other_gene
TWIST1	41	0.011401	0.290915	0	0.053215	Other_gene
ITPR3	39	0.011085	0.327419	0	0.039683	mRNA up
PTPRB	36	0.007233	0.37609	0.018487	0.029667	mRNA up

**Table 7:** The topological characteristics of the top 25 gene nodes in the PPI network of the green module within the GSE92681 dataset.

Name	Degree	Average shortest path length	Betweenness centrality	Closeness centrality	Clustering coefficient	Topological coefficient	Regulate
PSMA2	145	2.84572072	0.30956005	0.35140483	0	0.01436782	Other gene
IK	83	3.14752252	0.16898579	0.3177102	0	0.02628697	mRNA down
BCAR1	82	3.0731982	0.18704239	0.32539392	0	0.02264808	Other gene
TCF12	77	2.88400901	0.27221312	0.34673955	0	0.01974026	Other gene
SAV1	74	3.6768018	0.14809265	0.2719755	0	0.02402402	Other gene
NDN	61	3.29391892	0.12878792	0.30358974	0	0.02157032	Other gene
ERGIC3	55	3.31644144	0.11672478	0.30152801	0	0.02121212	Other gene
ASB3	38	3.51914414	0.07839935	0.28416	0	0.03827751	Other gene
MMP7	37	4.70157658	0.08120061	0.21269461	0	0.04054054	mRNA up
WT1	35	3.74887387	0.08035061	0.26674677	0	0.04642857	Other gene
DGCR14	32	3.37274775	0.05414526	0.29649416	0	0.05208333	Other gene
RIOK3	28	3.19256757	0.06657298	0.31322751	0	0.05844156	Other gene
DUS3L	26	3.3704955	0.04976931	0.29669228	0	0.05668016	Other gene
FAM74A4	26	4.65202703	0.02931155	0.21496006	0	0.19230769	Other gene
FAM74A6	25	4.65427928	0.0262998	0.21485604	0	0.19333333	Other gene
LRRC1	23	3.20157658	0.05583233	0.31234611	0	0.06126482	Other gene
PCYT2	21	3.31869369	0.04192798	0.30132338	0	0.05555556	Other gene
UBC	18	2.51351351	0.42421401	0.39784946	0	0.06050955	Other gene
BCORL1	16	3.4786036	0.02550435	0.28747167	0	0.08455882	Other gene
NCOA7	13	4.68581081	0.02298401	0.21341024	0	0.07692308	Other gene
STX10	13	3.48536036	0.02685944	0.28691438	0	0.07692308	Other gene
SGK3	12	3.35472973	0.03075664	0.29808661	0	0.11507937	Other gene
PRKX	11	4.67004505	0.01823525	0.2141307	0	0.18181818	Other gene
RNF135	10	4.73536036	0.01245585	0.21117717	0	0.2	Other gene
DFNA5	9	3.49436937	0.01078561	0.28617467	0	0.16339869	Other gene

**Table 8:** The topological characteristics of the top 20 nodes in the ceRNA network.

Name	Degree	Average shortest path length	Betweenness centrality	Closeness centrality	Topological coefficient
XIST	95	1.85534591	0.78103311	0.53898305	0.03168803
hsa-miR-340-5p	21	2.58490566	0.07287356	0.38686131	0.06802721
MIR155HG	17	3.73899371	0.06134578	0.26745164	0.09207161
hsa-miR-4731-5p	15	2.7327044	0.05609297	0.36593786	0.07777778
hsa-miR-5590-3p	15	2.71069182	0.03529256	0.36890951	0.09122807
hsa-miR-27a-3p	14	2.75157233	0.02751162	0.36342857	0.0924812
hsa-miR-27b-3p	14	2.75157233	0.02751162	0.36342857	0.0924812
hsa-miR-329-3p	14	2.71698113	0.03095743	0.36805556	0.09323308
hsa-miR-362-3p	13	2.72327044	0.02835696	0.36720554	0.09797571
LCP1	13	3.71069182	0.0012642	0.26949153	0.36153846
hsa-miR-494-3p	13	2.72955975	0.03199119	0.36635945	0.10576923
hsa-miR-424-5p	12	2.71698113	0.02811	0.36805556	0.12152778
hsa-miR-2682-5p	12	2.72327044	0.02462859	0.36720554	0.12457045
hsa-miR-515-5p	12	2.78301887	0.02610732	0.35932203	0.10283688
hsa-miR-766-5p	12	2.70440252	0.04407579	0.36976744	0.11139456
hsa-miR-449c-5p	12	2.72327044	0.01602592	0.36720554	0.13058419
EZH2	11	3.40880503	0.01905647	0.29335793	0.27548209
NR4A1	11	3.46226415	0.01590906	0.28882834	0.30976431
hsa-miR-34a-5p	11	2.77044025	0.01309547	0.36095346	0.13492823
hsa-miR-449a	11	2.77044025	0.01309547	0.36095346	0.13492823

## Data Availability

The data used to support the findings of this study are available from the corresponding author upon reasonable request.

## Supplementary Materials

Supplementary 1 File S1: the topological characteristics of all DEG nodes in the PPI network shown in Figure 4.

Supplementary 2 File S2: the topological characteristics of all nodes in the DElncRNA-DEG interaction network.

Supplementary 3 File S3: the topological characteristics of all nodes in the TF-DEG interaction network.

Supplementary 4 File S4: the topological characteristics of all DEG nodes in the PPI network for the significant blue module of GSE77459 dataset.

Supplementary 5 File S5: the topological characteristics of all DEG nodes in the PPI network for the significant green module of GSE92681 dataset.

Supplementary 6 File S6: the topological characteristics of all nodes in the ceRNA network.
